# Long-term outcome of sphincteroplasty with separate suturing of the internal and the external anal sphincter

**DOI:** 10.1007/s10151-019-02122-7

**Published:** 2019-11-26

**Authors:** M. R. Berg, H. Gregussen, Y. Sahlin

**Affiliations:** 1 Department of Colorectal Surgery, Innlandet Hospital Trust Hamar, Hamar, Norway; 2 Faculty of Medicine, Institute of Clinical Medicine, University of Oslo, Oslo, Norway; 3 Department of Colorectal Surgery, Innlandet Hospital Trust Hamar, Pb 4453, 2326 Hamar, Norway

**Keywords:** Anal incontinence, Obstetric anal sphincter injury, Sphincter repair

## Abstract

**Background:**

Sphincteroplasty is one of the treatment options for anal incontinence following obstetric injury. The aim of the study was to evaluate the long-term effect of sphincteroplasty with separate suturing of the internal and the external anal sphincter on anal continence.

**Methods:**

A retrospective study was conducted on women who had sphincteroplasty for treatment of anal incontinence following obstetric injury. Women operated between January 1, 2011 and December 31, 2014 at Sykehuset Innlandet Hospital Trust Hamar, were invited to answer a questionnaire and participate in a clinical examination, including endoanal sonography.

**Results:**

111 (86.7%) women participated. Median postoperative follow-up was 44.5 months, and 63.8% of the participants experienced an improvement of at least three points in the St. Mark’s incontinence score. Fecal urgency and daily fecal leakage persisted in 39.4% and 6.4% of the participants, respectively. The internal anal sphincter improvement persisted in 61.8% of the participants, and there was a median reduction of their St. Mark’s score of 6.0 points between the preoperative value and the value at long-term follow-up. There was no significant change in the St. Mark’s score of patients with persistent dehiscence of the internal anal sphincter.

**Conclusions:**

Sphincteroplasty, with separate suturing of the internal sphincter resulted in continence for stool maintained for at least 3 years in the majority of the patients, while there was an improvement in continence in nearly two-thirds.

## Introduction

Perineal laceration of the anal sphincters is a feared complication of vaginal delivery. The incidence of overt obstetric anal sphincter injuries is 0.5–5% [1, 2], but several studies have shown that sphincter injury occurs in 10–35% of all vaginal deliveries [3–7]. It is estimated that 33–59% of women with such injuries develop anal incontinence either shortly afterwards or several years later, compared to 13–27% of parous women without tears in the anal sphincter [8–12].

Anal incontinence after primary repair of obstetric anal sphincter injury occurs more frequently in women with persistent defects in the anal sphincter [13] and especially when the internal anal sphincter is also affected [14, 15]. One of the treatment options for anal incontinence caused by obstetric anal sphincter injury is sphincteroplasty, which aims to reestablish the normal anatomical structure and function of the anal sphincter complex.

Sphincteroplasty with overlapping repair technique, but without separate suturing of the internal anal sphincter, has previously been shown to give an improved short-term improvement in continence in 67–74% of patients [16–18]. Data on long-term outcome are sparse and most studies show attrition with time. A review article by Dudding et al. found that only 20% of patients were completely continent for stools 10 years postoperatively [5]. In a review article of 16 studies on the outcome of secondary sphincteroplasty by Glasgow and Lowry, most studies found that about 40% had good or excellent results at 5 years [19].

The aim of this study was to assess the long-term effect on continence of sphincteroplasty performed with separate end-to-end sutures of the internal sphincter and overlapping sutures of the external anal sphincter.

## Materials and methods

Invitations to participate in the study were sent by mail to all women who had sphincteroplasty after obstetric anal sphincter injury affecting both the internal and the external anal sphincter at Innlandet Hospital Trust Hamar between January 1, 2011 and December 31, 2014. The participants were identified through a search in the digital journal system for patients with the procedural ICD-10 code JHC10 (sphincteroplasty). Reminders were sent by mail 1 and 2 months after the initial invitation to all non-responders with known addresses.

Participants who had a colostomy or an implanted pacemaker for sacral nerve modulation at the time of long-term follow-up were excluded, because it would not be possible to determine whether their anal incontinence remained unchanged, worsened or improved after the sphincteroplasty.

Written, informed consent was obtained from all women who agreed to participate in the study.

**Fig. 1:**
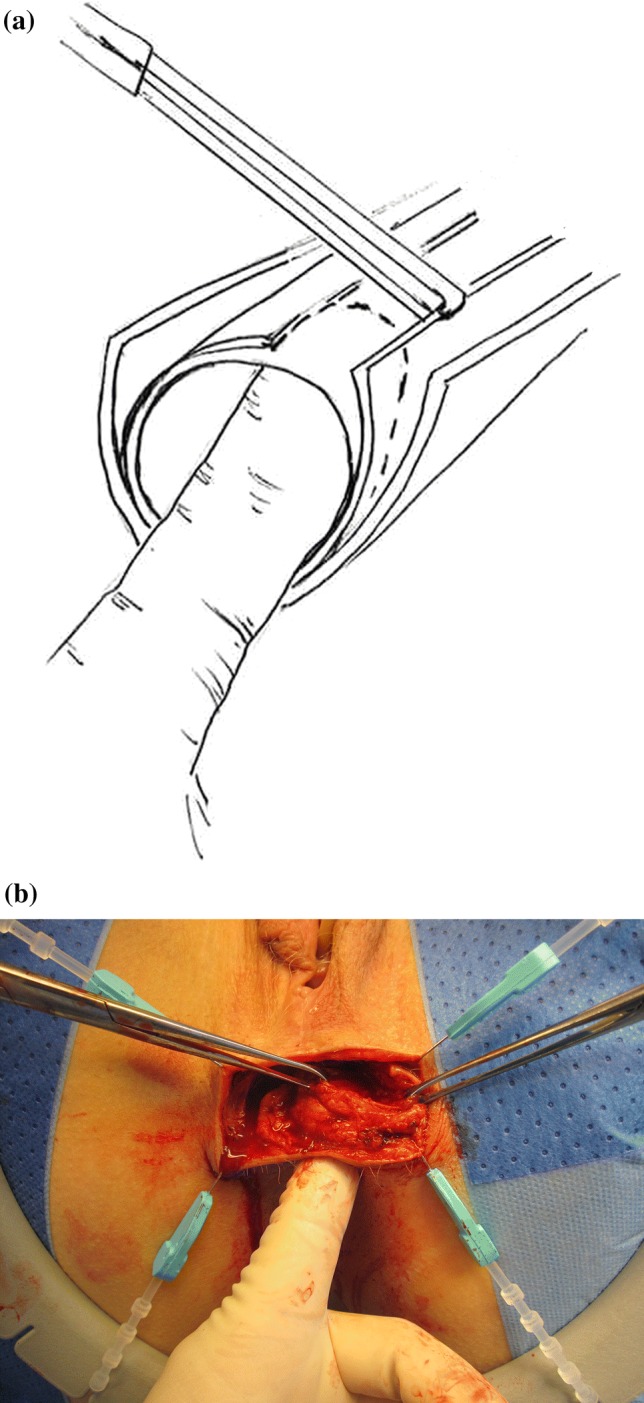
**a** Illustration showing how to identify the torn ends of the internal anal sphincter during surgery. By pulling with a forceps on the end of the internal sphincter while palpating with one finger in the anal canal, the internal anal sphincter was identified**. b** Perioperative photograph. By pulling with a forceps on the end of the sphincters while palpating with one finger in the anal canal, the torn ends of the muscle could be identified

The patients were placed in a lithotomy position and operated under general anesthesia, without muscle relaxation. A curvilinear incision was made transversely between the anus and the vaginal introitus. Scar tissue was dissected from the posterior vaginal wall and from the anterior anal canal. Lateral mobilization extended into the perianal fat pads. Adequate mobilization is necessary to ensure a tension-free wrap. The scar tissue in the midline was then divided. The gap between the external and internal anal sphincter was identified. By pulling with a forceps on the end of the internal sphincter while palpating with one finger in the anal canal, the internal anal sphincter was identified (Fig. 1). It is important to locate the gap of the internal sphincter with endoanal ultrasound prior to the surgery. This gives an idea of how far out to the sides the severed ends are to be found. Intraoperative endoanal ultrasound was not performed, due to swelling of the tissues the images provided were difficult to interpret.

The internal anal sphincter was sutured end-to-end with interrupted polydioxanone (PDS) 3-0 sutures. The external anal sphincter was sutured with overlapping interrupted PDS 3-0 sutures. The skin was closed with a T-suture using Monosyn 3-0. A small part of the skin was left open for drainage. A Foley catheter was used until the 1st postoperative day. Perioperative prophylactic antibiotics with methronidazol 1 g and doxycycline 400 mg intravenously were administered.

At least one senior colorectal surgeon with extensive experience in the surgical technique participated in all sphincteroplasties during the study period.

Demographic variables such as age, body mass index (BMI) and parity were collected in a questionnaire, as well as the St. Mark’s score (Table 1) [20]. We chose to use the St. Mark’s score because it is a well-established and objective measure of anal incontinence. The score has proved suitable for self-reporting of symptoms, while also correlated well with the patients’ perceptions of their symptoms’ severity [21, 22].

**Table 1:** St Mark’s incontinence score

	Never	Rarely	Sometimes	Weekly	Daily
Incontinence for solid stool	0	1	2	3	4
Incontinence for liquid stool	0	1	2	3	4
Incontinence for gas	0	1	2	3	4
Alteration of lifestyle	0	1	2	3	4

	No	Yes
Need to wear a pad or plug	0	2
Taking constipating medicines	0	2
Lack of ability to defer defecation for 15 min	0	4

*Never* no episodes in the past 4 weeks; *Rarely* 1 episode in the past 4 weeks; *Sometimes* > 1 episode in the past 4 weeks, but < 1 episode a week; *Weekly* ≥ 1 episodes a week in the past 4 weeks, but < 1 episode a day: *Daily* ≥ 1 episodes a day in the past 4 weeks. Add one score from each row and sum to a total. The minimum score is zero and equals perfect continence; the maximum score is 24 and equals complete incontinence

Clinical variables such as the date of the delivery that caused the injury, date of the sphincteroplasty, registered postoperative complications, preoperative St. Mark’s score, St. Mark’s score at 6 weeks postoperatively, and the degree of injury to the internal and the external anal sphincter preoperatively were retrieved from the digital journal system. Injury and dehiscence were defined as a defect at least 60° of the 360° circular form of the anal sphincters, in at least 50% of the muscle’s height, measured by endoanal ultrasound. The St. Mark’s scores preoperatively and at 6 weeks postoperatively were collected during consultations with colorectal surgeons at the outpatient clinic. The St. Mark’s scores at long-term follow-up were collected through the questionnaires mailed to the patients, which they completed independently without intervention from the authors.

**Fig. 2:**
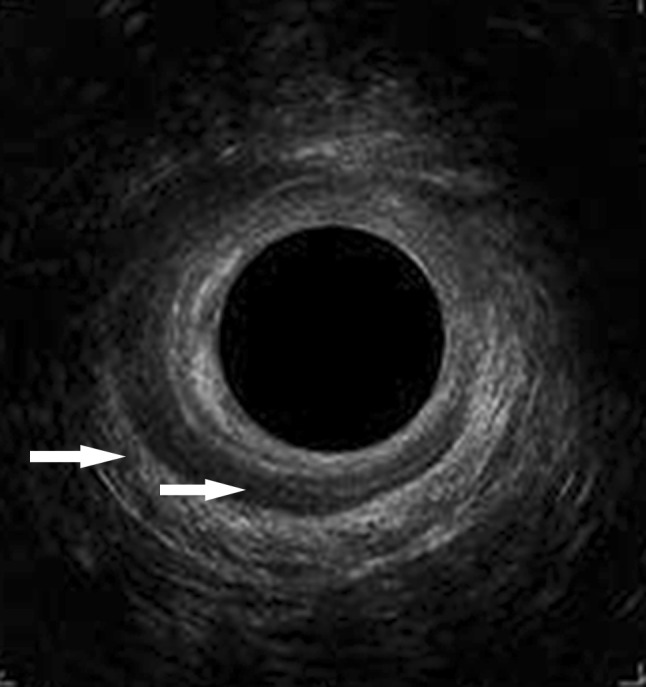
Endoanal ultrasound image. The hyperechoic outer circle is the external anal sphincter, and a defect is visible from the 9 o’clock position to the 3 o’clock position where the circle is discontinued and interrupted by a more hypoechoic area. The hypoechoic inner circle is the internal anal sphincter, and a defect is visible from the 11 o’clock position to the 4 o’clock position. Arrows point to each of the sphincters

All participants were invited to have a clinical examination, including endoanal ultrasound (Fig. 2). The pelvic floor was examined by inspection, palpation, and digital rectal exploration, during voluntary contraction of the pelvic floor and during relaxation. The anal sphincters were assessed by two-dimensional endoanal ultrasound (Bk Medical Flex Focus 800).

### Statistical analysis

SPSS package 24 was used to carry out the statistical analysis. We applied Student’s *t* test and Pearson’s Chi-squared test to compare means and proportions between different subgroups. Spearman’s correlation was used when unequal variances were assumed. *p* values of 0.05 or lower were considered significant.

The study protocol was approved by the Regional Ethical Committee of Medical Science in Norway (case number 344/16) and by the institutional review board at Innlandet Hospital Trust.

## Results

**Fig. 3:**
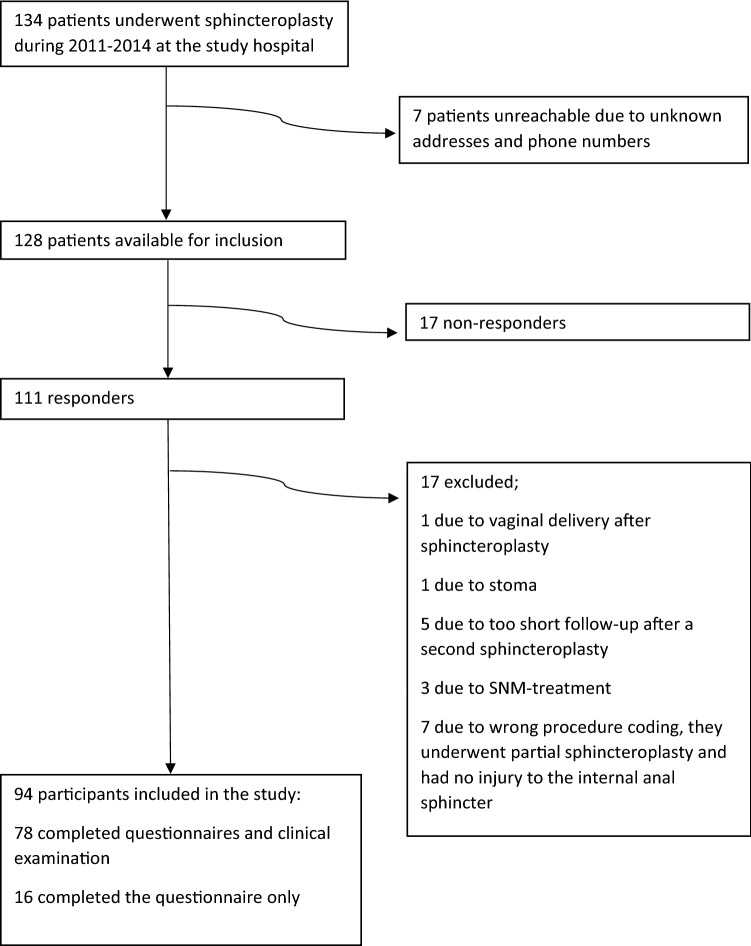
Flow chart of patient selection

Invitations to participate in the study were sent to 134 women. Seven were returned unopened due to unknown addresses. Of the remaining 128 women available for inclusion, 111 (86.7%) agreed to participate. Seventeen women were excluded (for the reasons outlined in Fig. 3). In total, 94 participants were included in the analysis, and 78 of these had endoanal ultrasound and clinical examination. Background variables are presented in Table 2.

**Table 2:** Patient data

*N *= 94	Median	Interquartile range	Range
Age (years)	43.0	11	25–77
Vaginal deliveries	2.0	2	1–6
Body mass index (kg/m^2^)	24.9	5.9	18.1–39.0
Years from injury to secondary surgery	8.5	12.5	0.5–46
Months of follow-up postoperatively	44.5	22	25–84

The St. Mark’s score improved by a median of 4.5 points at a median follow-up of 44.5 months (range 25–84 months). The median preoperative St. Mark’s score was 13 points, and 6.5 points at long-term follow-up. A paired-sample *t* test showed statistical significance for the difference between these values (*p *< 0.001) (Table 3).

**Table 3:** St. Mark’s score preoperatively, at 6 weeks postoperatively and at long-term follow-up

All participants (*N *= 94)	Mean	95% CI	Median	Interquartile range	Minimum–maximum
St. Mark’s score preoperatively	12.6	11.6 to 13.5	13.0	7.0	3 to 23
St. Mark’s score at 6 weeks postoperatively	3.7	2.9 to 4.6	2.5	6.0	0 to 19
St. Mark’s score at long-term follow-up	7.9	6.7 to 9.1	6.5	7.0	0 to 23
Reduction in preoperative St.Mark’s score at long-term follow-up, all participants (***N *= **94)^a^	4.7	3.5 to 5.9	4.5	7.0	− 12 to 19
Reduction in preoperative St.Mark’s score at long-term follow-up, internal anal sphincter adapted (***N *= **47)^a^	6.3	4.4 to 7.9	6.0	8.0	− 12 to 19
Reduction in preoperative St.Mark’s score at long-term follow-up, internal anal sphincter dehiscent (***N *= **29)^a^	0.5	− 2.1 to 2.9	2.0	5.0	− 11 to 12

^a^ In the rows showing the values for the reduction in preoperative St. Mark’s score long-term follow-up values, positive numbers indicates an improvement of anal continence and negative numbers indicate worsening

**Fig. 4:**
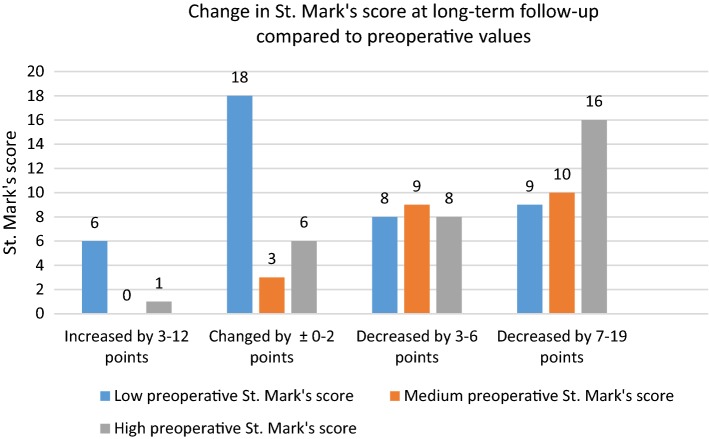
Change in St. Mark’s score at long-term follow-up compared to preoperative values. (1) Low preoperative St. Mark’s score defined as 3–11 points. (2) Medium preoperative St. Mark’s score defined as 12–15 points. (3) High preoperative St. Mark’s score defined as 16–24 points

An improvement of at least five points was achieved in 47 of the 94 participants [50.0% (95% CI 39.4–59.6)] when comparing the preoperative and follow-up St. Mark’s scores. Overall, 60 participants experienced an improvement in anal continence by at least three points. (Fig. 4).

Daily leakage of flatus was still present in 32 participants (34.0%) at long-term follow-up. Fecal leakage was experienced daily by 6 participants (6.4%) and weekly by 14 participants (14.9). Fecal urgency persisted in 37 participants (39.4%).

Among the 78 participants who had endoanal ultrasound at long-term follow-up, 64 (82.1%) attained successful repair of the external anal sphincter, while 4 (5.1%) had a complete dehiscence affecting the entire length of the external anal sphincter (Table 4). The difference between the preoperative St.Mark’s score and the score at long-term follow-up was not affected by the present status of the external anal sphincter (Tables 3, 4).

**Table 4:** Clinical examination and endoanal ultrasound findings at long-term follow-up

*N *= 78	N (%)	95% CI	Mean change in St. Mark’s score at long-term follow-up	95% CI	St. Mark’s score at long-term follow-up	95% CI
Intact internal anal sphincter	48 (61.5)	50.0 to 72.4	6.3	4.4 to 7.9	6.1	4.6 to 7.7
Incomplete^1^ rupture of internal anal sphincter	10 (12.8)	6.6 to 21.1	4.0	2.0 to 6.0	8.6	6.2 to 11.0
Complete^2^ rupture of internal anal sphincter	20 (25.6)	15.8 to 34.2	0.5*	− 2.1 to 2.9	13.0*	10.4 to 15.3
Intact external anal sphincter	64 (82.1)	72.4 to 89.5	4.0	2.4 to 5.4	8.2	6.7 to 9.7
Incomplete^a^ rupture of external anal sphincter	10 (12.8)	5.3 to 21.1	7.5	4.3 to 10.7	7.5	4.7 to 10.3
Complete^b^ rupture of external anal sphincter	4 (5.1)	1.3 to 10.5	5.8	0.5 to 13.2	9.5	4.3 to 14.3
Painful scar tissue in the perineal area^c^	24 (30.3)	19.7 to 40.8	5.1	3.0 to 7.6	8.5	6.5 to 10.5

^a^Incomplete rupture was defined as a visual gap at endoanal ultrasound of at least 60° of the full circumference of the anal sphincter, affecting at least 50% of the muscle’s length

^b^Complete rupture was defined as a visual gap at endoanal ultrasound of at least 60° of the full circumference of the anal sphincter, affecting the full length of the muscle

^c^Painful scar tissue was defined as visible scar tissue in the perineal area at clinical examination, combined with patient-reported discomfort or pain in the same area during intercourse or daily activities

At 6 weeks postoperatively, endoanal ultrasound verified successful healing of both the internal and the external anal sphincter in 90 of the 94 participants (95.7%). One additional participant attained successful healing of the internal anal sphincter, but not of the external which confirms that the internal anal sphincter was successfully identified during surgery in at least 91 of the 94 patients (96.8%).Two participants had dehiscence of both the internal and the external anal sphincter at 6 weeks postoperatively, and 1 participant failed to attend the 6-week follow-up visit. Median and mean extent (in degrees) of the tear in the anal sphincter preoperatively, at 6 weeks postoperatively and at long-term follow-up are presented in Table 5.

**Table 5:** Size of defect in the anal sphincters at endoanal ultrasound

All participants (*N *= 94)	Mean	95% CI	Median	Interquartile range	Minimum–maximum
Defect in internal sphincter preoperatively (°)	123	117–129	120	15.0	60–180
Defect in internal sphincter at 5 weeks postoperatively (°)	3	0–7.8	0	0.0	0–120
Defect in internal sphincter at long-term follow-up (°) (***N *= **78)	39	28–52	0	90.0	0–180
Defect in external sphincter preoperatively (°)	123	123–132	120	30.0	60–180
Defect in external sphincter at 6 weeks postoperatively(°)	3	0–7.8	0	0.0	0–120
Defect in external sphincter at follow-up(°) (***N *= **78)	19	11–129	0	7.5	0–120

Clock hours are also commonly used to describe the size of defects in the anal sphincters, and 1 h is equivalent to 30°

The internal anal sphincter remained apposed in 48 participants (61.5%), while 20 (25.6%) had a complete dehiscence affecting the entire length of the muscle (Table 4). There was no significant reduction of the St. Mark’s score of participants with persistent dehiscence of the internal anal sphincter [mean of 0.5 points improvement (95% CI − 2.1 to 2.9)] when comparing preoperative values to values at long-term follow-up. The participants with successful repair of the internal anal sphincter had a mean reduction of the St. Mark’s score of 6.3 points (95% CI 4.4–7.9). The difference in the change in the St. Mark’s score between these two groups was statistically significant (*p *= 0.001).

Postoperative wound infection was the most common complication after sphincteroplasty and occurred in 27 of the 94 participants (28.7%). Postoperative wound infections were associated with dehiscence of the internal anal sphincter, with a risk ratio of 1.99 (95% CI 1.22–4.79). Iatrogenic rectal or anal canal injury or postoperative fistula was not identified in any of the patients, however, three patients had fistula before the sphincteroplasty. In these cases, the sphincteroplasty was combined with repair of the fistula.

Time between the delivery that caused the injury and sphincteroplasty ranged from 5 months to 46 years (median 8.5 years). We used Spearman’s correlation for non-normally distributed variables and found no significant association (*p *= 0.78) between time between injury and sphincteroplasty and the change in St. Mark’s score at long-term follow-up.

Postmenopausal participants had a significantly higher St. Mark’s score before surgery than participants who had not reached menopause (15.3 points versus 11.6 points, *p *= 0.001). There was no significant difference between the postmenopausal participants and the non-menopausal participants as regards the change in the St. Mark’s score [5.2 (95% CI 3.1–7.5) versus 4.4 points (95% CI 3.0–5.8), *p *= 0.599].

There were no significant differences in the long-term outcome between the 15 participants operated on twice and the 79 participants operated on once (*p *= 0.68). The mean reduction of St. Mark’s score was 4.4 points (95% CI 0.5–8.2) in the 15 participants who had a second sphincteroplasty (Table 6). The mean reduction in the St. Mark’s score of the participants who had a single sphincteroplasty was 4.7 points (95% CI 3.5–5.0). All the 15 participants operated on twice had experienced dehiscence of the anal sphincter shortly after the first sphincteroplasty.

**Table 6:** Data for participants operated on twice

*N *= 15	Median	Interquartile range	Range
Age (years)	45.0	8.0	33.0 to 55.0
Vaginal deliveries	2.0	1.0	1.0 to 5.0
Body mass index (kg/m^2^)	25.9	4.3	21.6 to 32.0
Years from injury to secondary surgery	9.0	11.0	1.0 to 32.0
Months of follow-up postoperatively	48.0	23.0	26.0 to 84.0
St. Mark’s score preoperatively	16.0	8.0	4.0 to 21.0
St. Mark’s score at 6 weeks	4.0	6.0	0.0 to 15.0
St. Mark’s score at long-term follow-up	8.0	13.0	0.0 to 23.0
Reduction in preoperative St. Mark’s score at long-term follow-up	5.0	14.0	− 11.0 to 19.0

**Fig. 5:**
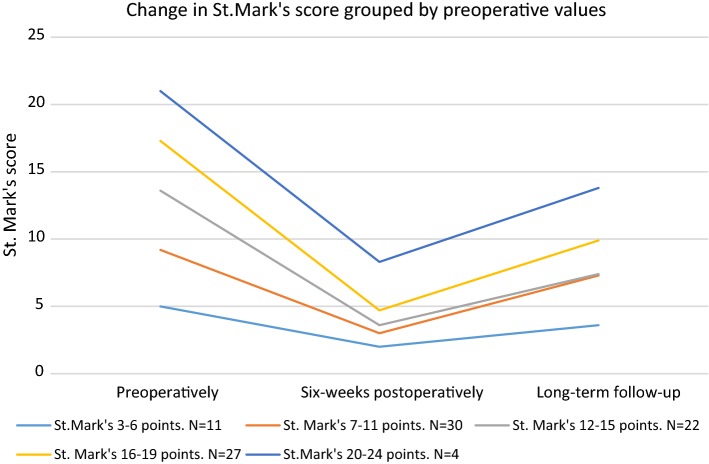
Change in St. Mark’s score grouped by preoperative values

The preoperative St. Mark’s score impacted the outcome at long-term follow-up. The 53 participants with a preoperative St. Mark’s score higher than 11 achieved a mean reduction of the St. Mark’s score greater than that reported by the 41 participants with preoperative St. Mark’s score of 11 or lower (*p *< 0.0001) (Fig. 5).

## Discussion

The findings of this study indicate that sphincteroplasty with separate suturing of the external and the internal anal sphincter improves anal continence in about two-thirds of patients with obstetric anal sphincter injuries when evaluated ≥ 2 years postoperatively. About half of the participants were completely continent for stool, and only a quarter of them experienced fecal leakage on a weekly basis. Less than half experienced fecal urgency. Persistent defects in the internal anal sphincter were associated with a poorer outcome of the sphincteroplasty.

Strengths of the study were a high response rate, and high number of participants examined with endoanal ultrasound, as well as a reasonably large study population as compared to previous studies on sphincteroplasty. All participants underwent sphincteroplasty with the same technique.

The main limitation of the study is that it was not a randomized controlled trial between different surgical techniques. Nine of the 111 women who agreed to participate were excluded from the analysis, because they had had treatment with a stoma, sacral nerve modulation or a second sphincteroplasty less than 12 months prior to the study. It must be assumed that the results of the first sphincteroplasty these nine women had were suboptimal, and their exclusion probably leads to an overestimation of the effect of sphincteroplasty.

Previous studies on long-term outcome after sphincter repair without separate suturing of the internal sphincter have shown that 48–65% of patients maintain good results at follow-up 3–10 years postoperatively [16–18, 23–26]. The definition of good results varies between studies, as shown by Glasgow et al. [19], and this complicates comparisons between different methods and studies. Some studies report any improvement of anal incontinence as “good” or “fair” [17, 18]. In other studies, the definitions are based on whether the patients are continent for liquid and solid stools [16, 23, 24].

Johnson et al. presented the outcomes of 33 women at a median of 103 months of follow-up after secondary anterior sphincteroplasty with the overlap technique. The median preoperative St. Mark’s score was 12 and the score at follow-up was 9 [17]. Our study showed a greater improvement of anal continence, with a median preoperative St. Mark’s score of 13, and a median score at long-term follow-up of 6.5. Johnson et al. had a longer follow-up time, which could explain the difference, as anal incontinence is known to deteriorate with time after sphincteroplasty [18, 19, 23, 27–29]. On the other hand, it is important to note that separate suturing of the internal and the external anal sphincters was not performed in the patients in the above-mentioned study, and this could also explain why our study showed a better outcome.

Zorcolo et al. assessed 62 patients with anal incontinence due to obstetric injury at a median of 70 months after anterior sphincteroplasty with the overlap technique. Overall, 70% of their participants achieved an improvement in anal continence, and the median St. Mark’s score decreased from 18 points before the sphincteroplasty to 11 at follow-up [18]. In a study including 120 women after a median of 111 months from anterior overlapping sphincteroplasty, Karoui et al. found that 37% maintained continence for stools, and an additional 23% achieved improvement in anal continence [25] Both these studies present similar findings to ours, and the size of the study populations is also similar.

The most common complication after sphincteroplasty was postoperative wound infection, which was experienced by about one-third of the participants. Zorcolo et al. and Karoui et al. found that wound infection occurred in 20–26% of patients following sphincteroplasty, and Karoui showed that infections were associated with a poorer outcome [18, 25]. In our study, postoperative wound infections were associated with dehiscence of the internal anal sphincter (risk ratio 1.99). Postoperative wound infection was associated with a poorer continence, and the same applied to persistent dehiscence of the internal anal sphincter. This is consistent with the findings of previous studies, where persistent defects in the internal anal sphincter were found to be associated with higher degrees of anal incontinence [13–15, 25]. We did not find the same association between the status of the external anal sphincter and the St. Mark’s score at long-term follow-up. This indicates that healing and apposition of the internal anal sphincter are important to achieve improvement of anal continence, and wound infection is a risk factor for dehiscence and poorer outcome of the sphincteroplasty.

In this study, the St. Mark’s score at 6 weeks postoperatively could not predict the long-term outcome of sphincteroplasty.

Among the 94 participants included in the study, 15 had previously had an unsuccessful secondary sphincteroplasty with verified dehiscence of the anal sphincters on ultrasound seen a few months postoperatively. A comparison between these 15 participants and the 79 participants with only 1 sphincteroplasty showed that both groups achieved the same improvement regarding anal continence. This confirms the findings of Giordano et al. [30]. Reoperation should be considered in patients with confirmed suture rupture and dehiscence of the anal sphincters shortly after the first sphincteroplasty, as many of them achieve improved anal continence after repeat sphincteroplasty.

The present study found that patients with higher St. Mark’s scores preoperatively achieved better results at long-term follow-up. Patients with preoperative St. Mark’s scores less than 12 achieved only minor improvement of their anal incontinence. This study included women with low preoperative degrees of anal incontinence, which we are aware is controversial. The purpose of offering sphincteroplasty to this subgroup was not primarily to alleviate symptoms of anal incontinence, but to reconstruct normal anatomy and alleviate sexual impairment caused by lack of a normal perineal body and a thin posterior vaginal wall.

Our findings indicate that secondary sphincteroplasty should not be offered to patients with low degrees of anal incontinence unless the purpose of the surgery is to alleviate other symptoms such as impaired sexual function due to abnormal anatomy. Patients should also be warned that anal incontinence could actually deteriorate after sphincteroplasty if their preoperative St. Mark’s score is low.

Several studies have shown that only 10–16% of women suffering from anal incontinence seek help on their own initiative [9, 12, 31–33]. To ensure that patients with suboptimal outcomes after sphincteroplasty get access to other treatment options, we recommend annual postoperative follow-up consultations for at least 2 years.

## Conclusions

Our study shows that sphincteroplasty with separate suturing of the internal sphincter results in continence for stools maintained for at least 3 years among the majority of the patients and an improvement of anal continence in nearly two-thirds of the patients. In our opinion, sphincteroplasty should have a role in the treatment of anal incontinence after obstetric anal sphincter injuries. Adequate patient selection is important to achieve good outcomes, and annual follow-up for the first few years postoperatively is important to ensure patients with suboptimal outcomes get access to other treatment options.

## References

[1] Ampt AJ, Patterson JA, Roberts CL, Ford JB (2015). Obstetric anal sphincter injury rates among primiparous women with different modes of vaginal delivery. Int J Gynaecol Obstet.

[2] Laine K, Gissler M, Pirhonen J (2009). Changing incidence of anal sphincter tears in four Nordic countries through the last decades. Eur J Obstet Gynecol Reprod Biol.

[3] Guzman Rojas RA, Shek KL, Langer SM, Dietz HP (2013). Prevalence of anal sphincter injury in primiparous women. Ultrasound Obstet Gynecol.

[4] Andrews V, Sultan AH, Thakar R, Jones PW (2006). Occult anal sphincter injuries–myth or reality?. BJOG.

[5] Dudding TC, Vaizey CJ, Kamm MA (2008). Obstetric anal sphincter injury: incidence, risk factors, and management. Ann Surg.

[6] Oberwalder M, Connor J, Wexner SD (2003). Meta-analysis to determine the incidence of obstetric anal sphincter damage. Br J Surg.

[7] Sultan AH, Kamm MA, Hudson CN, Thomas JM, Bartram CI (1993). Anal-sphincter disruption during vaginal delivery. N Engl J Med.

[8] Cornelisse S, Arendsen LP, van Kuijk SM, Kluivers KB, van Dillen J, Weemhoff M (2016). Obstetric anal sphincter injury: a follow-up questionnaire study on longer-term outcomes. Int Urogynecol J.

[9] Gjessing H, Backe B, Sahlin Y (1998). Third degree obstetric tears; outcome after primary repair. Acta Obstet Gynecol Scand.

[10] Pollack J, Nordenstam J, Brismar S, Lopez A, Altman D, Zetterstrom J (2004). Anal incontinence after vaginal delivery: a five-year prospective cohort study. Obstet Gynecol.

[11] Sultan AH, Kamm MA, Hudson CN, Bartram CI (1994). Third degree obstetric anal sphincter tears: risk factors and outcome of primary repair. BMJ.

[12] Wagenius J, Laurin J (2003). Clinical symptoms after anal sphincter rupture: a retrospective study. Acta Obstet Gynecol Scand.

[13] Oude Lohuis EJ, Everhardt E (2014). Outcome of obstetric anal sphincter injuries in terms of persisting endoanal ultrasonographic defects and defecatory symptoms. Int J Gynaecol Obstet.

[14] Fornell EU, Matthiesen L, Sjodahl R, Berg G (2005). Obstetric anal sphincter injury ten years after: subjective and objective long term effects. BJOG.

[15] Norderval S, Oian P, Revhaug A, Vonen B (2005). Anal incontinence after obstetric sphincter tears: outcome of anatomic primary repairs. Dis Colon Rectum.

[16] Barisic GI, Krivokapic ZV, Markovic VA, Popovic MA (2006). Outcome of overlapping anal sphincter repair after 3 months and after a mean of 80 months. Int J Colorectal Dis.

[17] Johnson E, Carlsen E, Steen TB, Backer Hjorthaug JO, Eriksen MT, Johannessen HO (2010). Short- and long-term results of secondary anterior sphincteroplasty in 33 patients with obstetric injury. Acta Obstet Gynecol Scand.

[18] Zorcolo L, Covotta L, Bartolo DC (2005). Outcome of anterior sphincter repair for obstetric injury: comparison of early and late results. Dis Colon Rectum.

[19] Glasgow SC, Lowry AC (2012). Long-term outcomes of anal sphincter repair for fecal incontinence: a systematic review. Dis Colon Rectum.

[20] Vaizey CJ, Carapeti E, Cahill JA, Kamm MA (1999). Prospective comparison of faecal incontinence grading systems. Gut.

[21] Maeda Y, Pares D, Norton C, Vaizey CJ, Kamm MA (2008). Does the St. Mark’s incontinence score reflect patients’ perceptions? A review of 390 patients. Dis Colon Rectum.

[22] Roos AM, Sultan AH, Thakar R (2009). St. Mark’s incontinence score for assessment of anal incontinence following obstetric anal sphincter injuries (OASIS). Int Urogynecol J Pelvic Floor Dysfunct.

[23] Bravo Gutierrez A, Madoff RD, Lowry AC, Parker SC, Buie WD, Baxter NN (2004). Long-term results of anterior sphincteroplasty. Dis Colon Rectum.

[24] Halverson AL, Hull TL (2002). Long-term outcome of overlapping anal sphincter repair. Dis Colon Rectum.

[25] Karoui S, Leroi AM, Koning E, Menard JF, Michot F, Denis P (2000). Results of sphincteroplasty in 86 patients with anal incontinence. Dis Colon Rectum..

[26] Lamblin G, Bouvier P, Damon H (2014). Long-term outcome after overlapping anterior anal sphincter repair for fecal incontinence. Int J Colorectal Dis.

[27] Londono-Schimmer EE, Garcia-Duperly R, Nicholls RJ, Ritchie JK, Hawley PR, Thomson JP (1994). Overlapping anal sphincter repair for faecal incontinence due to sphincter trauma: five year follow-up functional results. Int J Colorectal Dis.

[28] Malouf AJ, Norton CS, Engel AF, Nicholls RJ, Kamm MA (2000). Long-term results of overlapping anterior anal-sphincter repair for obstetric trauma. Lancet.

[29] Kuismanen K, Nieminen K, Karjalainen K, Lehto K, Uotila J (2018). Outcomes of primary anal sphincter repair after obstetric injury and evaluation of a novel three-choice assessment. Tech Coloproctol.

[30] Giordano P, Renzi A, Efron J (2002). Previous sphincter repair does not affect the outcome of repeat repair. Dis Colon Rectum.

[31] Evers EC, Blomquist JL, McDermott KC, Handa VL (2012). Obstetrical anal sphincter laceration and anal incontinence 5–10 years after childbirth. Am J Obstet Gynecol.

[32] Lo J, Osterweil P, Li H, Mori T, Eden KB, Guise JM (2010). Quality of life in women with postpartum anal incontinence. Obstet Gynecol.

[33] Norderval S, Nsubuga D, Bjelke C, Frasunek J, Myklebust I, Vonen B (2004). Anal incontinence after obstetric sphincter tears: incidence in a Norwegian county. Acta Obstet Gynecol Scand.

